# Utilizing Deep Machine Learning for Prognostication of Oral Squamous Cell Carcinoma—A Systematic Review

**DOI:** 10.3389/froh.2021.686863

**Published:** 2021-07-26

**Authors:** Rasheed Omobolaji Alabi, Ibrahim O. Bello, Omar Youssef, Mohammed Elmusrati, Antti A. Mäkitie, Alhadi Almangush

**Affiliations:** ^1^Department of Industrial Digitalization, School of Technology and Innovations, University of Vaasa, Vaasa, Finland; ^2^Research Program in Systems Oncology, Faculty of Medicine, University of Helsinki, Helsinki, Finland; ^3^Department of Oral Medicine and Diagnostic Science, College of Dentistry, King Saud University, Riyadh, Saudi Arabia; ^4^Department of Pathology, University of Helsinki, Helsinki, Finland; ^5^Department of Otorhinolaryngology – Head and Neck Surgery, University of Helsinki and Helsinki University Hospital, Helsinki, Finland; ^6^Division of Ear, Nose and Throat Diseases, Department of Clinical Sciences, Intervention and Technology, Karolinska Institutet and Karolinska University Hospital, Stockholm, Sweden; ^7^Institute of Biomedicine, Pathology, University of Turku, Turku, Finland; ^8^Faculty of Dentistry, University of Misurata, Misurata, Libya

**Keywords:** machine learning, deep learning, oral cancer, prognostication, systematic reveiw

## Abstract

The application of deep machine learning, a subfield of artificial intelligence, has become a growing area of interest in predictive medicine in recent years. The deep machine learning approach has been used to analyze imaging and radiomics and to develop models that have the potential to assist the clinicians to make an informed and guided decision that can assist to improve patient outcomes. Improved prognostication of oral squamous cell carcinoma (OSCC) will greatly benefit the clinical management of oral cancer patients. This review examines the recent development in the field of deep learning for OSCC prognostication. The search was carried out using five different databases—PubMed, Scopus, OvidMedline, Web of Science, and Institute of Electrical and Electronic Engineers (IEEE). The search was carried time from inception until 15 May 2021. There were 34 studies that have used deep machine learning for the prognostication of OSCC. The majority of these studies used a convolutional neural network (CNN). This review showed that a range of novel imaging modalities such as computed tomography (or enhanced computed tomography) images and spectra data have shown significant applicability to improve OSCC outcomes. The average specificity, sensitivity, area under receiving operating characteristics curve [AUC]), and accuracy for studies that used spectra data were 0.97, 0.99, 0.96, and 96.6%, respectively. Conversely, the corresponding average values for these parameters for computed tomography images were 0.84, 0.81, 0.967, and 81.8%, respectively. Ethical concerns such as privacy and confidentiality, data and model bias, peer disagreement, responsibility gap, patient-clinician relationship, and patient autonomy have limited the widespread adoption of these models in daily clinical practices. The accumulated evidence indicates that deep machine learning models have great potential in the prognostication of OSCC. This approach offers a more generic model that requires less data engineering with improved accuracy.

## Introduction

A total of 377, 713 new cases of oral cavity and lip cancer and 177, 757 deaths related to oral cancer were reported in the year 2020 [1]. Considering the location of oral squamous cell carcinoma (OSCC) and the corresponding aggressive behavior of this disease, it has been reported to have significant effects on the patients' post-treatment quality of life [2]. Recently, clear advances in diagnostic techniques and treatment modalities have been achieved [3]. However, OSCC is still characterized by a low average survival rate [4]. Accurate prognostication remains of utmost importance to improve survival rates [5].

Traditionally, the treatment of cancer depends mainly on tumor staging. However, staging discrepancies have contributed to inaccurate prognostication in OSCC patients [2]. Despite the increasing number of prognostic markers, the overall prognosis of the disease has not changed significantly [6]. This may be due to the challenges in the integration of these markers in the current staging system [7, 8]. Additionally, individualized treatment of patient on a case-by-case basis is lacking. Therefore, improved diagnostic and prognostic accuracy could significantly assist the clinicians in making informed decisions regarding appropriate treatment for better survival [9].

To this end, machine learning techniques (shallow learning) have been reported to offer improved prognostication of OSCC [9, 10]. Of note, the use of machine learning has been reported to provide a more accurate prognostication than the traditional statistical analyses [9, 11–14]. Machine learning techniques have been able to show promising results because they are able to discern the complex relationships between the variables contained in the dataset [9]. Considering the touted feasibility and benefits of the machine learning techniques in cancer prognostication, its application in this field has attracted significant attention in recent years. This is because it is poised to assist the clinicians in making informed decisions thereby improving and promoting better management of patient health. Interestingly, the advancements in technology have led to the modification of shallow machine learning to deep machine learning. This deep learning approach has also been touted to improve cancer management.

In this study, we aim to systematically review the published studies that have utilized deep machine learning techniques for OSCC prognostication. This is necessary to show the state-of-the-art performance of deep learning analytic methods for prognostication of the disease. Thus, the focused question was: “Does deep machine learning technique play a role in improving prognostication accuracy and guiding clinicians in making an informed decision.”

## Methods

### Search Protocol

Detailed literature searches were performed using databases such as OvidMedline, PubMed, Scopus, Web of Science, and Institute of Electrical and Electronics Engineers (IEEE) from their inception until 15 May 2021. RefWorks software was used to properly manage the potentially relevant articles and remove any duplicate articles. Additionally, the reference lists of the included articles were manually searched to ensure that all the relevant articles have been included.

### Search Strategy

The search approach was developed by combining search keywords: [((“oral cancer” OR “oral squamous cell carcinoma” OR “pre-cancerous” OR “oral potentially malignant”) AND (“deep learning”))].

### Eligibility Criteria

#### Inclusion Criteria

The Population, Exposure, Comparator, Outcomes, and Study design (PECOS) framework was used to define the research question(s) of this review. Thus, the P in the PECOS framework represents population (patients) with OSCC; E depicts that deep machine learning has been applied for prognostication, C ensures that the parameter of interest have OSCC patients with or without this parameter, O indicates that there is a clear outcome to be determined by the deep learning techniques, and S indicates that observational studies and/or clinical trials were also considered. Thus, original, observational, and clinical trials that utilized deep learning techniques for prognostication in OSCC were included. Additionally, only studies published in the English language were considered.

#### Exclusion Criteria

Studies in languages other than English and those that did not utilize deep learning for prognostication in oral cancer were excluded. Case reports, editorials, surveys, book chapter, comparative papers, symposium articles, conference articles, short communications, abstracts, opinions, perspectives, invited reviews, and letters to the editor were also excluded.

### Study Selection

The study selection process was carried out in two distinct phases. Firstly, the titles and abstracts of potentially relevant articles were examined after the removal of duplicates. This phase was conducted by two independent reviewers (R.A., & O.Y.). A data extraction sheet was used for this process to ensure proper documentation with a Cohen's Kappa coefficient (κ = 0.91) for inter-observer reliability. This stage was followed by consensus meeting and discussion to resolve possible discrepancies before the study could be included in this review. For the second phase, these two independent reviewers extracted relevant information relating to the study characteristics of each of these potentially relevant articles.

### Parameters Extracted

The independent reviewers (R.A., & O.Y.) extracted the following information from each of the included studies; author (s) name, year of publication, country, oral cancer description, study objectives, sample population, type of data used, performance of the deep learning model, and conclusions. This information is presented in Table 1.

**Table 1:** Extracts of the main findings from the included studies.

**Authors, country, (site)**	**No of cases/images**	**Machine learning methods**	**Data types used in the deep learning**	**Study aim**	**Results**	**Performance metric(s)**	**Conclusion**
Shams and Htike [15], Malaysia, (Oral cavity)	86	Traditional machine learning classifiers: Support Vector Machine (SVM), Regularized Least Squares (RLS) & Multi-layer perceptron (MLP). Deep learning method: Deep Neural Network (DNN).	Gene expression data	Prediction of the risk of oral cancer in patients with oral premalignant lesion (OPL).	The DNN method outperformed the traditional classifier.	Sensitivity:0.98 Specificity: 0.94 Accuracy: 96.5%	The possibility of oral cancer was predicted with high accuracy in patients with oral premalignant lesion.
Aubreville et al. [16], Germany, (Oral cavity)	7,894	Deep learning method: CNN.	Anatomical images	To detect oral cancer.	The deep learning method was able to detect on image (Confocal laser endomicroscopy images of oral squamous cell carcinoma [OSCC]).	Sensitivity: 0.86 Specificity: 0.90 Accuracy: 88.3% AUC: 0.96	The deep learning offered automatic detection of oral cancer for effective management of the cancer.
Uthoff et al. [17], USA, (Oral cavity)	170	Deep learning method: CNN.	Intraoral images	To distinguish between precancerous and cancerous lesions early.	Automatic and affordable smartphone-based system for oral screening distinction.	Sensitivity: 0.85 Specificity: 0.88	Effective management of oral cancer through early detection.
Das et al. [18], India, (Oral cavity)	126	Deep learning method: CNN	Histological images	To diagnose oral squamous cell carcinoma through the automatic identification of relevant regions.	The regions were identified with relatively high accuracy.	Accuracy: 96.9%	The identified region ensured the effective diagnosis of oral squamous cell carcinoma.
Song et al. [19], India and USA (Oral cancer)	190	Deep learning method: CNN	Auto-fluorescence images	To screen high-risk populations for oral cancer in low and middle income countries	The deep learning approach was able to differentiate between dysplasia and malignancy tissue from the normal ones	Sensitivity: 0.850 Specificity: 0.887 Accuracy: 86.9%	The approach showed effective means in classifying dual-modal images for oral cancer detection.
Yan et al. [20], China, TSCC	22	Deep learning method: CNN	Raman Spectroscopy	To differentiate between tongue squamous cell carcinoma tissue from non-tongue squamous cell carcinoma tissue.	This approach showed a novel method for classifying spectral data of tongue squamous cell carcinoma and normal tissue using fiber optic Raman spectroscopy and ensemble CNN model.	Sensitivity: 0.992 Specificity: 0.992	The combination of Raman spectroscopy and CNN offer the possibility of intraoperative evaluation of the tongue squamous cell carcinoma.
Yan et al. [20], China, TSCC	24	Deep learning method: CNN	Raman Spectroscopy	To differentiate between tongue squamous cell carcinoma tissue from non-tongue squamous cell carcinoma tissue.	The deep learning showed promising results between the tongue squamous cell carcinoma and non-tongue squamous cell carcinoma tissue regions.	Sensitivity: 0.9907 Specificity: 0.9537	The combination of Raman spectroscopy and CNN offer the possibility of intraoperative evaluation of the tongue squamous cell carcinoma.
Yu et al. [21], China, OTSCC	36	Traditional machine learning classifier: Principle Component Analysis (PCA), Support Vector Machine (SVM), and Linear Discriminant Analysis (LDA) Deep learning method: CNN	Raman spectral data	To differentiate between oral tongue squamous cell carcinoma from non-tongue squamous cell carcinoma.	The CNN outperformed the traditional classifiers.	Sensitivity: 0.99 Specificity: 0.94 Accuracy: 96.9%	The combination of Raman spectroscopy and CNN offer the possibility of distinction between oral tongue squamous cell carcinoma from non-tongue squamous cell carcinoma.
Chan et al. [22], Taiwan, (Oral cavity)	80	Deep learning method: CNN	Auto-fluorescence images	The detection of oral cancer.	The Gabor filter provided a useful feature extraction to accurately detect oral cancer.	Sensitivity: 0.93 Specificity: 0.94	The oral cancer was successfully detected.
Sunny et al. [23], India, Germany, and America, (Oral cavity)	100	Artificial Neural Network (ANN)	Cytological images	Prediction of the risk of oral cancer in patients with oral premalignant lesion (OPL).	The ANN showed higher accuracy.	Specificity: 0.90 Accuracy: 86.0%	The tele-cytology approach offered remote and effective method to detect patients with oral premalignant lesion.
Jeyaraj and Samuel Nadar, [24], India (Oral cavity)	100	Deep learning method: CNN	Computed tomography images	To distinguish between cancerous tumor with benign and cancerous tumor with normal tissue.	The CNN showed significant performance to detect cancerous tumor with benign.	Sensitivity: 0.94 Specificity: 0.91 Accuracy: 91.4%	This approach provide early detection and effective management of oral cancer.
Ariji et al. [25], Japan, (Oral cavity)	441	Deep learning method: CNN	Computed tomography images	To diagnose lymph node metastasis.	The CNN showed performance that is comparable to the pathologists.	Sensitivity: 0.75 Specificity: 0.81 Accuracy: 78.2%.	The CNN showed a promising result to revolutionize oral cancer management.
Xu et al. [26], China, (Oral cavity)	~7,000	Three-Dimensional Convolutional Neural Networks (3DCNN)	Computed tomography images	To distinguish between benign and malignant oral cancers.	The 3DCNN variant outperformed the 2DCNN.	Accuracy: 75.4%	The 3DCNN properly distinguished between benign and malignant oral cancer.
Kim et al. [2], Korea, OSCC	255	Traditional machine learning classifiers: Random survival forest, Cox proportional hazard model (CPH). Deep learning method: CNN	Clinicopathologic images	Oral cancer survival prediction in patients.	The deep learning method performed better than the traditional machine learning methods.	C-index : 0.781	Survival prediction can offer a good approach to properly manage oral cancer.
Das et al. [27], India, OSCC	126	Deep learning method: CNN	Histological images	To use nucleus detection and segmentation to diagnose OSCC.	The deep learning network was able to use computer-aided tool for automatic detection and delineation of the detected nucleus from oral histological images.	Sensitivity: 0.8887 Specificity: 0.8203	This approach is aimed at assisting clinicians in the OSCC diagnosis.
Shaban et al. [28] (OSCC)	~70	Deep learning method: CNN	Clinical image	To propose an automated method for the objective quantification of tumor-infiltrating lymphocytes	The deep learning approach accurately quantified the tumor-infiltrating lymphocytes which provided high prognostic value for staging and accurate predictor of disease progression.	Accuracy: 96.13%	The quantification of tumor-infiltrating lymphocytes is capable of providing vital information about prognosis to the clinicians.
Jeyaraj et al. [29], India (Oral cavity)	>25	Deep learning method: Deep Boltzmann Machine	Hyperspectral images	To detect and classify oral cancer.	The deep Boltzmann network was able to classify normal, pre- and post-cancerous regions.	Accuracy: 94.75%	The proposed digital pre-screening framework using deep learning classifier fusion on hyperspectral thermal imaging provides higher potential for cancer identification.
Panigrahi et al. [30], India, (OSCC)	150	Deep learning methods: Capsule network	Histopathological images	To identify oral cancer in histopathological images	The capsule network showed promising results as an automated tool for classification of oral cancer	Sensitivity: 0.9778 Specificity: 0.9692 Accuracy: 97.35%	The capsule network is suitable to identify histopathological images in early-stage oral cancer.
Kouznetsova et al. [31], USA, (Oral cancer)	180	Deep learning methods	Saliva metabolites	To distinguish between oral cancer and periodontitis using machine learning.	The deep learning approach offer the possibility to distinguish between periodontal disease and oral cancer using deep learning.	Accuracy: 79.54%	It gives the opportunity of a non-invasive, quick, and effective method of oral cancer diagnosis.
Das et al. [32], India, OSCC	156	Deep learning method: CNN	Histopathological images	To propose a CNN-based multi-class grading method of oral squamous cell carcinoma.	The deep learning offer an effective grading system.	Accuracy: 92.15%	The CNN can be used to diagnose patients with oral squamous cell carcinoma.
Ariji et al. [33], Japan (Oral Squamous Cell Carcinoma)	703	Deep learning method: CNN	Computed tomography images	To clarify computed tomography diagnostic performance in extranodal extension of cervical lymph node metastases.	The deep learning method outperformed the radiologists.	Accuracy: 84.0%	Extranodal extension can be properly diagnosed using deep learning.
Ariji et al. [33], Japan OSCC	365	Deep learning method: CNN	Computed tomography images	To detect cervical lymph nodes metastasis	The DetectNet was suitable for object detection.	Sensitivity: 0.73	A system to automatically detect cervical lymph nodes metastasis.
Xia et al. [34], China OTSCC	24	Deep learning method: CNN	Fiber optic Raman spectroscopy	To detect oral tongue squamous cell carcinoma.	Deep learning an effective method for oral tongue squamous cell carcinoma resection margins.	Sensitivity: 0.995 Specificity: 0.995 AUC: 0.9996	The deep learning showed accuracy that is comparable to the state of the art methods.
Fujima et al. [35], USA and Japan, (OCSCC)	113	Deep learning method: CNN	Computed tomography images	To predict disease free survival (DFS) in patients with OCSCC	The deep learning showed show improved rate for disease free survival.	Sensitivity: 0.8 Specificity: 0.8 Accuracy: 0.8	The deep learning approach may predict treatment outcome in patient with OCSCC
Fu et al. [36], China (OCSCC)	44,409	Deep learning method: CNN	Clinical images	To identify patients with OCSCC	This automated approach provides rapid and non-invasive detection of OCSCC.	Sensitivity: 0.896 Specificity: 0.806 AUC: 0.935	The performance of the deep learning is comparable to an expert and better than medical student.
Ding et al. [37], China, (TSCC)	22	Deep learning method: Deep residual network	Raman spectral data	To distinguish between TSCC from non-cancerous.	The deep residual network is able to offer accurate detection of TSCC	Sensitivity: 0.9738 Specificity: 0.9875 Accuracy: 98.25%	The deep learning technology can be used to classify TSCC tissues.
Jubair et al. [38], Jordan and Greece (Oral cancer)	716	Deep learning method: CNN	Clinical image	To classify clinical image into either benign and malignant or oral potentially malignant disorder (OPML)	The deep learning offer an effective and low-budget means of oral cancer screening	Sensitivity: 0.867 Specificity: 0.845 Accuracy: 85.0%	Deep learning can improve the quality of oral cancer screen and early detection
Welikala et al. [39], Multicenter, (Oral cancer)	1,085	Deep learning method: CNN	Pathological images	To detect oral lesions early through the automated detection and classification with deep learning.	Deep learning methods build automated systems.	F1 score: 87.1%	Deep learning has the potential to detect oral lesions.
Paderno et al. [40], Italy, (Oral cancer)	34	Deep learning method: CNN	Neoplastic images	To segment squamous cell carcinoma (SCC) of the cavity.	The deep learning showed a promising approach in segmenting squamous cell carcinoma.	Dice similarity coefficient (Dsc) mean value: 0.6559	The deep learning application offers a real time application in the diagnosis of oral cancer.
Tomita et al. [41] (OSCC)	320	Deep learning method: CNN	Computed tomography images	To differentiate between benign and metastatic cervical lymph nodes	The pretreated contrast-enhanced computed tomography trained with deep learning outperformed the radiologists	AUC: 0.967	The deep learning tool is posited as a diagnostic tool for differentiating between benign and metastatic cervical lymph nodes.
Nanditha et al. [42], India, (Oral cancer)	320	Deep learning method: Ensemble deep learning	Oral lesion images	To classify lesions as either precancerous or normal lesions.	Automated diagnostic tool based on deep learning showed promising performance in classifying between precancerous and normal lesions	Sensitivity: 0.9814 Specificity: 0.9423 Accuracy: 96.2%	Modification to deep learning can further improve its importance in cancer management.
Musulin et al. [43], Croatia (OSCC)	322	Deep learning method: CNN	Histopathological images	Deep learning approach to automatically grade OSCC and segment epithelial stroma tissue.	The deep learning based on stationary wavelet transform (SWT) was able to automatically grade OSCC into multiclass.	AUC: 0.963 FI score: 0.955	The deep learning based model has the potential in the diagnosis of OSCC.
Trajanovski et al. [44], Netherlands (OTSCC)	14	Deep learning method: CNN	Hyperspectral image data	To detect tongue cancer using deep learning semantic segmentation	The approach showed that important information regarding tumor decision is encoded in various channels, but some channel selection and filtering is beneficial over the full spectra.	AUC: 0.924	The hyperspectral imaging combined with deep learning is poised to offer promising alternatives to improving cancer management.
Kim et al. [45], Korea (Oral cancer)	173	Deep learning method: CNN	Gene expression + Clinical data	To group the patients into risk groups	The importance of tumor-infiltrating lymphocytes was emphasized in tumor microenvironment	Accuracy: 97.2%	The patients' survival pattern was successfully predicted.

*AUC, Area Under Receiving Operating Characteristics (ROC) curve; CNN, Convolutional Neural Network; TSCC, Tongue Squamous Cell Carcinoma; OTSCC, Oral Tongue Squamous Cell Carcinoma; OSCC, Oral Squamous Cell Carcinoma; OCSCC, Oral Cavity Squamous Cell Carcinom. Similarly, when the performance metrics were reported differently for training and validation, only the validation performance metrics was considered*.

### Quality Assessment of the Included Studies

**Figure 1:**
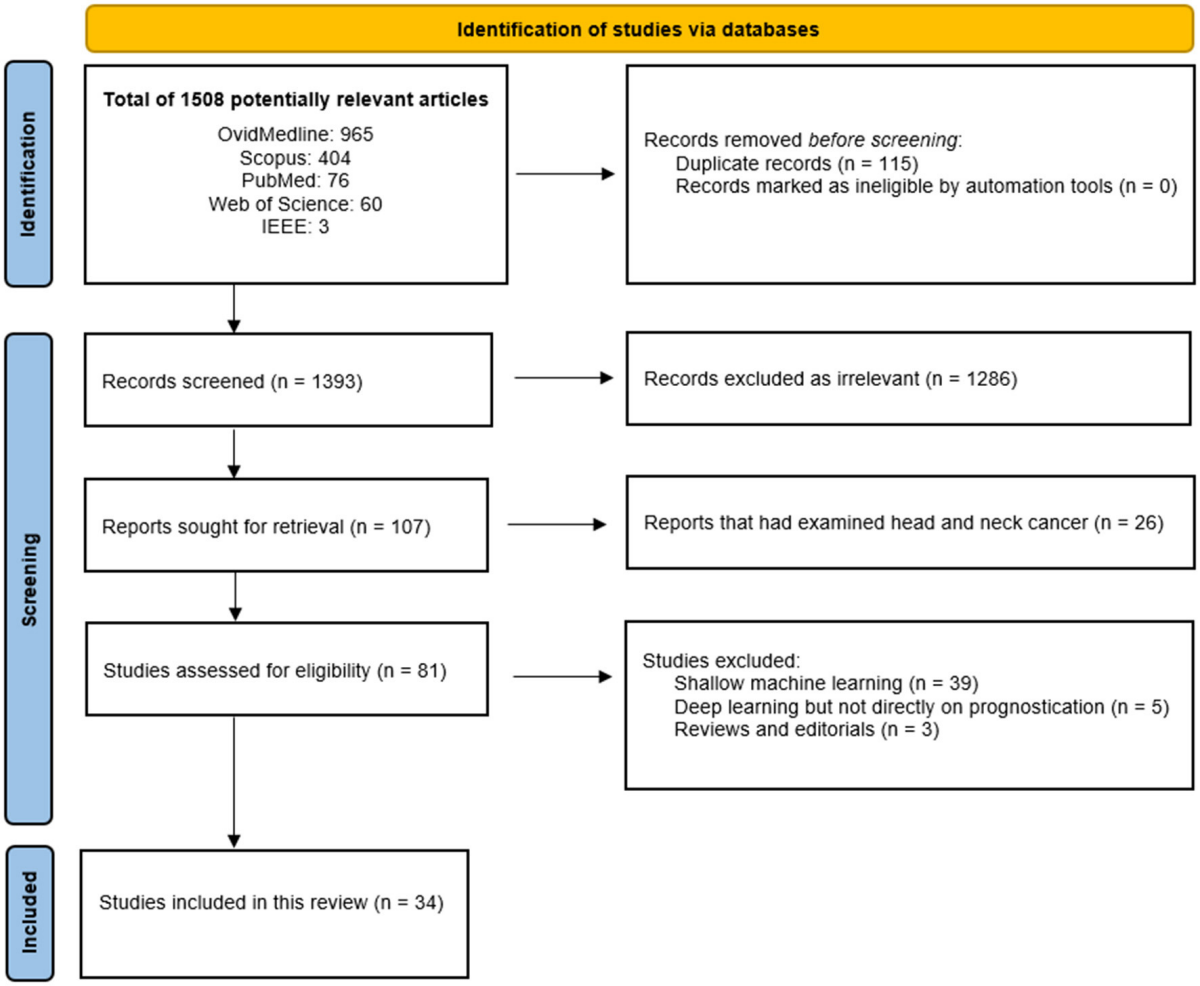
The PRISMA flow chart for the included studies [46].

The Preferred Reporting Items for Systematic Review and Meta-Analysis (PRISMA) methodology was used to document the searching and screening processes in this study (Figure 1) [46, 47]. The quality of the included studies were accessed using the Prediction model Risk of Bias Assessment Tool (PROBAST) as shown in Table 2. Additionally, PROBAST was used to evaluate and assess the risk of bias (ROB) of the potential studies to be included in this review. As the study examines deep machine learning method, the predictors' parameter from the PROBAST tool was modified to include the robustness of the methodology used in the included studies.

**Table 2:** The presentation of PROBAST results.

**Study**	**ROB**				**Applicability**			**Overall**	
	**Participants**	**Predictors**	**Outcome**	**Analysis**	**Participants**	**Predictors**	**Outcome**	**ROB**	**Applicability**
Shams and Hitke [15]	+	?	+	+	+	?	+	–	–
Aubreville et al. [16]	+	+	+	+	+	+	+	+	+
Uthoff et al. [17]	+	+	+	+	+	+	+	+	+
Das et al. [18]	+	+	+	+	+	+	+	+	+
Song et al. [19]	+	+	+	+	+	+	+	+	+
Yan et al. [20]	+	+	+	+	+	+	+	+	+
Yan et al. [20]	+	+	+	+	+	+	+	+	+
Yu et al. [21]	+	+	+	+	+	+	+	+	+
Chan et al. [22]	+	+	+	+	+	+	+	+	+
Sunny et al. [23]	+	+	+	+	+	+	+	+	+
Jeyaraj and Nadar [24]	+	+	+	+	+	+	+	+	+
Ariji et al. [25]	+	+	+	+	+	+	+	+	+
Xu et al. [26]	+	+	+	+	+	+	+	+	+
Kim et al. [2]	+	+	+	+	+	+	+	+	+
Das et al. [27]	+	+	+	+	+	+	+	+	+
Shaban et al. [28]	+	+	+	+	+	+	+	+	+
Jeyaraj et al. [29]	–	+	+	+	–	+	+	–	–
Panigrahi et al. [30]	+	+	+	+	+	+	+	+	+
Kouznetsova et al. [31]	+	+	+	+	+	+	+	+	+
Das et al. [32]	+	+	+	+	+	+	+	+	+
Ariji et al. [33]	+	+	+	+	+	+	+	+	+
Ariji et al. [33]	+	+	+	+	+	+	+	+	+
Xia et al. [34]	+	+	+	+	+	+	+	+	+
Fujima et al. [35]	+	+	+	+	+	+	+	+	+
Fu et al. [36]	+	+	+	+	+	+	+	+	+
Ding et al. [37]	+	+	+	+	+	+	+	+	+
Jubair et al. [38]	+	+	+	+	+	+	+	+	+
Welikala et al. [39]	+	+	+	+	+	+	+	+	+
Paderno et al. [40]	+	+	+	+	+	+	+	+	+
Tomita et al. [41]	+	+	+	+	+	+	+	+	+
Nanditha et al. [42]	+	+	+	+	+	+	+	+	+
Musulin et al. [43]	+	+	+	+	+	+	+	+	+
Trajanovski et al. [44]	+	+	+	+	+	+	+	+	+
Kim et al. [45]	+	+	+	+	+	+	+	+	+

*PROBAST, Prediction model Risk Of Bias Assessment Tool; ROB, Risk of Bias*.

*+Indicates Low ROB/Low concern regarding applicability*.

*−Indicates High ROB/high concern regarding applicability*.

*?Indicates unclear ROB/unclear concern regarding applicability*.

## Results

### Results of the Database Search

A total of 34 studies met the eligibility criteria and were included in this review [2, 15–45, 48]. The details of the study selection process have been described using the PRISMA flowchart (Figure 1) [46]. The included studies that utilized deep learning for prognostication of OSCC are summarized in Table 1. These studies concluded that the deep learning techniques could offer assistance to the clinicians in making informed decisions regarding choosing treatment options to avoid under-treatment or unnecessary treatments and thus achieving better management of the disease.

### Studies Characteristics of Relevant Studies

The majority of these studies used a convolutional neural network (CNN) [2, 15–22, 24–26, 28, 31–36, 38–41, 43–45, 48, 49]. Several data types such as gene expression data [15, 45], spectra data [20, 21, 29, 34, 37, 44, 48], and other image data types—anatomical [16], intraoral [17], histology [18, 27], auto-fluorescence [19, 22], cytology-image [23], neoplastic [40], clinical [28, 36, 38], oral lesions [42], computed tomography images [24–26, 33, 35, 41, 49], clinicopathologic [2], saliva metabolites [31], histopathological [30, 32, 43], and pathological [39] images have been used in the included studies.

Considering the reported performance metrics (specificity, sensitivity, and accuracy) and the accumulated evidence presented in the included studies, deep machine learning models have great potential in the prognostication of OSCC. This approach offers a more generic model that requires less data engineering with improved accuracy.

A single study reported the performance of deep learning with four different performance metrics (sensitivity, specificity, accuracy, and area under receiving operating characteristics curve [AUC]) [16]. Similarly, a total of 11 studies reported the combination of the trio of sensitivity, specificity, and accuracy as the performance metrics for the deep machine learning method [15, 19–21, 24, 25, 30, 35, 37, 38, 42]. Both specificity and sensitivity were used to depict the performance of the model [17, 20, 22, 27, 48]. Additionally, specificity and accuracy were also used to demonstrate the performance of the deep learning model for prognostication in OSCC [23]. Other studies used either accuracy, C-index (concordance index), F1-score, or Dice similarity coefficient (Dsc) mean value as the performance metrics for reporting the potential benefits of the deep learning model [2, 18, 18, 26, 28, 29, 31–33, 39–41, 44, 45, 49].

Most of these studies used either spectra data or computed tomography images [20, 21, 24–26, 29, 33–35, 37, 41, 44, 48, 49]. The average specificity, sensitivity, area under receiving operating characteristics curve [AUC]), and accuracy for studies that used spectra data were 0.97, 0.99, 0.96, and 96.6%, respectively. Conversely, the corresponding average values for these parameters for computed tomography images were 0.84, 0.81, 0.967, and 81.8%, respectively.

The deep machine learning method has been reported to show promising results in the prediction and detection of OSCC [2, 15, 16, 18, 19, 22–24, 27–30, 34, 36, 39, 44] and lymph node metastasis [25, 33, 41]. Furthermore, the deep machine learning model have been reported to show significant prognostication of grading of the disease [32, 43] and survival prediction of oral cancer patients [2, 35]. Additional, the ability of deep machine learning to differentiate between precancerous (potentially malignant) lesions and OSCC [15, 17, 23, 26, 38, 39, 42], as well as between the disease and, for example, periodontitis disease [31] has been highlighted in different studies. Also, deep machine learning models have shown touted benefits that differentiate between oral tongue squamous cell carcinoma (OTSCC) and non-tumorous tissue [20, 21, 26, 37, 48].

### Quality Assessment of the Studies Included in the Review

According to the PROBAST assessment, most (91.2%) of the included studies showed an overall low risk of bias and also exhibited low concern regarding applicability (Table 2).

## Discussion

In this systematic review, the utilization of deep machine learning for prognostication in oral squamous cell carcinoma was examined. The deep learning methodology had been used to analyze various types of medical data such as clinicopathologic, histopathologic, gene expression, image, Raman spectroscopy, saliva metabolites, and computed tomography for better prognostication in OSCC. This review showed that a range of novel imaging modalities such as computed tomography (or enhanced computed tomography) images and spectra data have shown significant applicability to improve OSCC outcomes. Hence, deep machine learning methodology combined with medical imaging data can offer better and improved prognostication of OSCC. This can significantly assist the clinical management of patients with the disease [50].

The performance of the deep learning technique was mostly reported with either the combination of sensitivity, specificity, and accuracy or using a single performance metric. Based on the reported accuracy of the deep machine learning techniques in the included studies, it is evident that the deep machine learning technique can play a significant role toward the improved prognostication of oral cancer and guide clinicians in making informed decisions. The approach of using deep learning for prognostication can provide low-cost screening [19, 36], smartphone-based solution [17, 23], deep learning-based automatic prognostication [18, 27, 32], and early detection and prediction of outcomes [15, 17, 23, 24, 37–39, 42].

The utilization of deep machine learning for prognostication include the distinction between potentially malignant disease and OSCC [15, 17, 26, 39], differentiation between the oral tongue squamous cell carcinoma from non-tumorous tissue [20, 21, 34], prediction and detection of oral squamous cell carcinoma [16, 18, 22–24, 51], diagnosis of lymph node metastasis [25, 33], differentiation between OSCC and diseases such as periodontitis [31], ability to offer multi-class grading of OSCC [32], and prediction of survival in OSCC patients [2].

The afore-mentioned diagnostic ability and prognostication can greatly benefit the clinical management of OSCC patients [50]. For instance, deep learning can assist the pathologists in the effective multi-class grading, thereby, assisting in the timely and effective treatment protocol for the patients [32]. This can reduce operational workload and the possibility of burnout for the pathologists and enhance the proper management of the disease through timely grading [32]. Similarly, the deep learning model is capable of stratifying the patients into high-risk patients where they could be assigned to a more aggressive regimen or low-risk where more conservative treatment may be enough. This informed decision could assist in the overall survival of these patients by reducing the possibility of side effects such as hormonal disorder, trismus, or dental disease [52, 53].

The availability of medical data in different formats (multi-omics data—genomic, expression, proteomic, transcriptomic, and clinicopathologic data) through various databases such as the cancer genome atlas (TCGA), gene expression omnibus (GEO) has emerged as a great challenge to the traditional statistical methods of cancer prognostication [50]. Additionally, with the increase in computational power, advancement in technology (neural network model architecture), availability of medical dataset, the widely used shallow machine learning techniques have been modified to produce a deep machine learning technique, also known as the deep neural network (DNN) [54]. Interestingly, shallow machine learning has been reported to show promising results in various prognostication tasks such as prediction of locoregional recurrences [9, 10], survival [55], occult nodal metastasis [56], and performed better than other methods such as nomograms [57].

**Figure 2:**
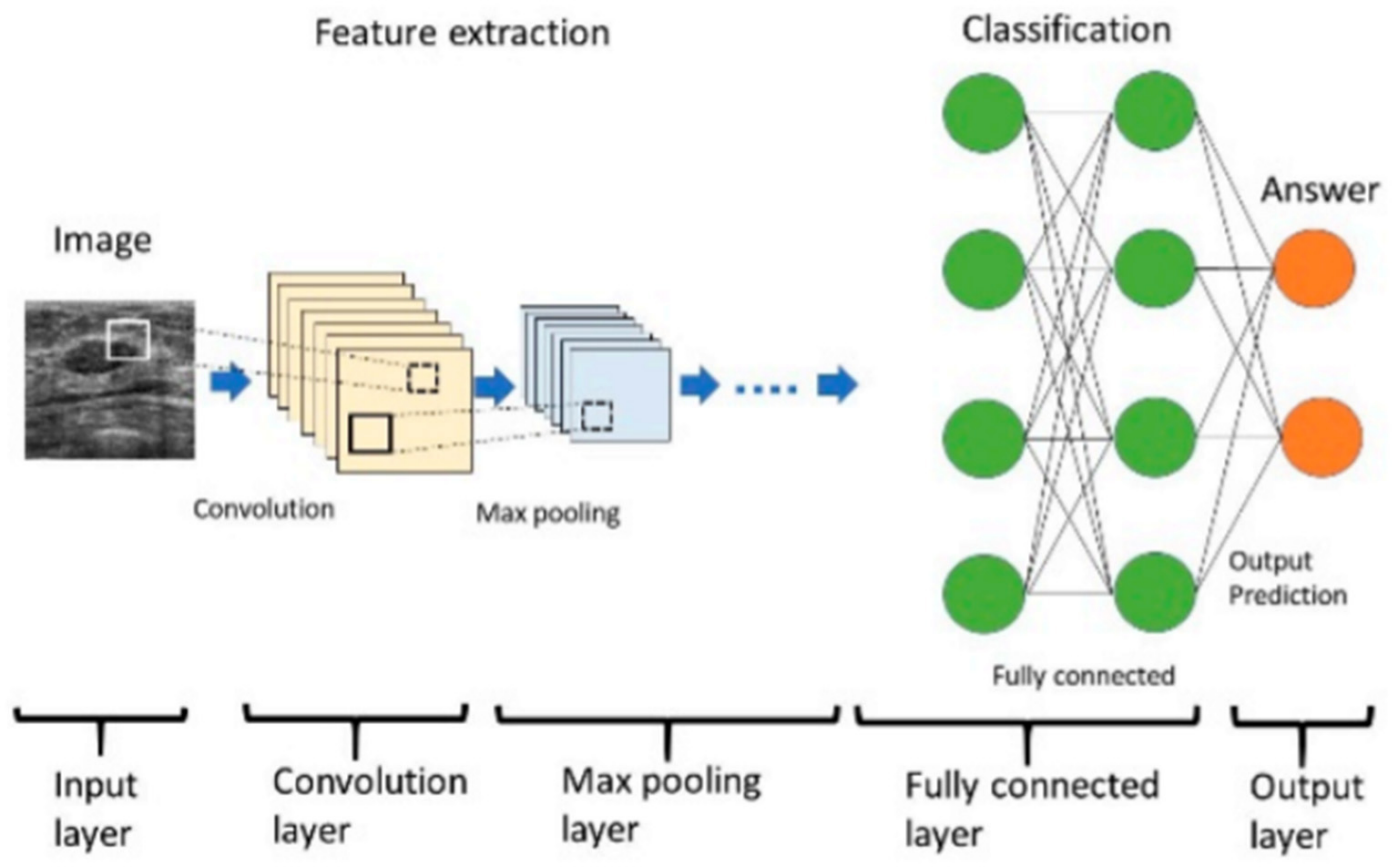
The architecture of a convolutional neural network [62].

Despite these promising results by the shallow machine learning techniques, the deep machine learning techniques have been reported to perform equally or outperform the shallow machine learning method [50, 58, 59] as it is more flexible, requires less feature engineering, and consist of complex layers and multiple neurons in each layer [50, 60, 61] (Figure 2). This gives deep machine learning a better predictive power [50]. An example of the deep neural network commonly used in cancer prognostication is the convolutional neural network (CNN) which is usually used for medical image data [50] (Figure 2). In CNN, the convolution and max-pooling layer are responsible for feature extraction of the input data [62] (Figure 2). While the convolution layers facilitate feature extraction from the image data, the pooling layers ensure that overfitting is minimized. The results from the convolution and pooling layers are passed to the fully connected layer for classification into labels (output) [62]. Apart from the CNN, the recurrent neural network (RNN) is another type of deep neural network which is suitable for text and sequence data [50].

In spite of the prospects of the deep learning models to improve OSCC outcomes through improved detection and diagnosis, most of these models have found widespread adoption in daily clinical practices. Several reasons have been attributed to the limited use of these models in clinical practice. A recent study showed that ethical concerns limited the potential use of these models in actual practices [63]. These ethical concerns include privacy and confidentiality, data and model bias, peer disagreement, responsibility gap, patient-clinician relationship, and patient autonomy [63]. Similarly, a recent study by Alabi et al., highlighted the concerns that are either inherent to the science of machine learning (technical) or the actual clinical implementation [64]. These include black box concern, amount of data, interpretability, explainability, and generalizability [64].

The strength of this systematic literature review is that it specifically examined the published studies that had examined deep learning in OSCC. This approach ensured that the contribution of the state-of-the-art deep learning techniques in OSCC was specifically examined. In addition, it offers the opportunity to understand the future research avenue of the application of deep learning in OSCC. An example of an exciting research area would be the development of new data fusion algorithms for improved prognostication in disease.

The main limitation is that most of the included studies used different performance metrics for the evaluation of the deep learning techniques. Similarly, the deep learning techniques used different data types in the analyses. Thus, it was challenging to make an insightful conclusion on the performance of these deep learning technique. Additionally, the dataset used to train the model was relatively small in most of the studies. Most of the developed deep learning models in the published studies were not externally validated. The study by Alhadi et al., provided an update on staging and World Health Organization grading as reliable OSCC prognostic indicators [65]. To the best of our knowledge, there is a dearth of published studies that have examined the application of machine learning for staging. Therefore, this serves as a potential area of further research in the future.

In conclusion, there is an increase in the application of deep learning for prognostication in OSCC. The deep learning models are poised to predict cancer prognosis more accurately. Thereby, offering precision and personalized management of the disease. It has shown to be better or equivalent to the current approaches in daily clinical practices. It is expected that the deep learning techniques can assist in the proper management of OSCC through improved diagnostic performance, insightful clinical decision making, streamline clinicians' work, offer a potential to reduce cancer care costs in the screening, and an effective assessment and surveillance of the disease. Thus, the clinicians and patients can spend more time in communication and in making shared decisions to improve the quality of care. In the future, it is important to develop deep learning models that combine multiple datasets from multiple modalities.

## Summary Points

### What Was Already Known on the Topic

There are several published studies on the application of machine learning techniques to analyze oral squamous cell carcinoma (OSCC).

### What Knowledge This Study Adds

This study systematically reviewed the published studies that examined the application of deep machine learning techniques for prognostication in OSCC.

The majority of these studies used a convolutional neural network (CNN).

This review showed that a range of novel imaging modalities such as computed tomography (or enhanced computed tomography) images and spectra data have shown significant applicability to improve oral cancer outcomes.

The average specificity, sensitivity, area under receiving operating characteristics curve [AUC]), and accuracy for studies that used spectra data were 0.97, 0.99, 0.96, and 96.6%, respectively. Conversely, the average specificity, sensitivity, AUC, and accuracy for computed tomography images were 0.84, 0.81, 0.967, and 81.8%.

The study concluded that the deep learning techniques could offer assistance to the clinicians in making informed decisions regarding choosing treatment options to avoid under-treatment or unnecessary treatments for the better management of OSCC.

## Data Availability Statement

The original contributions generated for the study are included in the article/supplementary material, further inquiries can be directed to the corresponding author/s.

### Protocol and Registration

The databases of the International Prospective Register of Systematic Reviews (PROSPERO) were searched for any registered protocols on a similar topic to this systematic review. The protocol on the methodology of this review was therefore submitted to PROSPERO for a registration protocol number.

## Author Contributions

RA, ME, AA, and AM: study concepts and study design and data analysis and interpretation. RA and OY: studies extraction. IB, OY, and AA: acquisition and quality control of included studies. RA, OY, AA, and AM: manuscript preparation. AM and IB: manuscript review. AA, RA, and IB: manuscript editing. All authors approved the final manuscript for submission.

## Conflict of Interest

The authors declare that the research was conducted in the absence of any commercial or financial relationships that could be construed as a potential conflict of interest.

## Publisher's Note

All claims expressed in this article are solely those of the authors and do not necessarily represent those of their affiliated organizations, or those of the publisher, the editors and the reviewers. Any product that may be evaluated in this article, or claim that may be made by its manufacturer, is not guaranteed or endorsed by the publisher.
